# Psychological Morbidity in IBD: The Dominant Role of Disease Activity over Subtype and Demographic Factors

**DOI:** 10.3390/jcm14124179

**Published:** 2025-06-12

**Authors:** Sinem Azizoglu, Idris Kurt, Huseyin Ahmet Tezel

**Affiliations:** 1 Department of Internal Medicine, Kocaeli City Hospital, 41060 İzmit, Turkey; sinem.oguz@hotmail.com; 2 Department of Gastroenterology, Trakya University School of Medicine, 22130 Edirne, Turkey; dr.atezel@gmail.com

**Keywords:** inflammatory bowel disease, ulcerative colitis, Crohn’s disease, anxiety, depression, disease activity, Hospital Anxiety and Depression Scale (HADS)

## Abstract

**Background**: Patients with inflammatory bowel disease (IBD) experience disproportionately high rates of anxiety and depressive symptoms, representing a 2–3-fold increased risk versus the general population. While psychological morbidity is well-documented, the relative contributions of disease activity (active vs. inactive), IBD subtype (ulcerative colitis [UC] vs. Crohn’s disease [CD]), and sociodemographic factors remain clinically contested. This study aimed to quantify these relationships to guide targeted interventions. **Methods**: We conducted a cross-sectional, single-center study comparing 94 consecutive IBD patients (61 UC, 33 CD; mean age 34.2 ± 11.1 years) with 79 age- and sex-matched healthy controls. The psychological status was assessed using the validated Turkish version of the Hospital Anxiety and Depression Scale (HADS), with 14 items (7 per subscale, 0–21 range) and established cutoffs (≥10 for anxiety, ≥7 for depression). Disease activity was objectively classified: UC patients via the Truelove–Witts Index (inactive, mild, moderate, or severe) and CD patients via the Crohn’s Disease Activity Index (CDAI < 150 inactive, ≥150 active). Statistical analyses employed SPSS 20.0 (IBM Corp., Armonk, NY, USA), including Student’s *t*-tests, Mann–Whitney U tests, and chi-square tests, with *p* < 0.05 as the significance threshold. **Results**: Disease activity was the strongest predictor: active IBD patients exhibited 64% higher anxiety symptom scores (11.2 ± 3.1 vs. 6.8 ± 2.4, *p* < 0.001) and 107% higher depressive symptom scores (8.9 ± 2.7 vs. 4.3 ± 1.9, *p* < 0.001) than inactive patients. Gender and education modulated the risk: females showed 23% elevated anxiety (OR 1.23, 95% CI 1.06–1.43; *p* = 0.008), while college education correlated with 31% lower depression odds (OR 0.69, 95% CI 0.51–0.93; *p* = 0.026). Strikingly, 32.1% of IBD patients met depressive symptom thresholds versus 17.7% of controls (*p* = 0.028). No UC/CD subtype differences emerged (*p* > 0.05). **Conclusions**: Our findings confirm that active IBD inflammation—not the disease subtype (UC/CD)—is the strongest predictor of anxiety and depressive symptoms, with one in three patients meeting clinical depression thresholds. Women and those with less education face heightened risks, underscoring the need for routine mental health screening during flares and targeted psychosocial support. These results advocate for integrated psychiatric care in IBD management to address this invisible burden.

## 1. Introduction

Inflammatory bowel diseases (IBDs), mainly encompassing ulcerative colitis (UC) and Crohn’s disease (CD), are chronic, relapsing conditions characterized by gastrointestinal inflammation [1]. UC manifests as superficial mucosal inflammation, typically originating in the rectum and extending contiguously to the colon, with alternating periods of exacerbation and remission. In contrast, CD may involve any segment of the gastrointestinal tract, from the oral cavity to the anus, and is distinguished by patchy, transmural inflammation that often leads to complications such as strictures or fistulae [2]. While these conditions can occur at any age, diagnosis peaks between 15 and 30 years—a pivotal life stage for education, career establishment, and relationships. Disease symptoms during this period can impair social functioning, heightening susceptibility to anxiety and depression. The unpredictable disease course, fear of flares, and potential surgical interventions further exacerbate psychological distress, fostering pessimism about long-term outcomes.

Chronic illnesses, including IBD, are strongly linked to elevated rates of depression and anxiety [3]. Graff et al. [4] reported that IBD patients exhibit higher anxiety and depression scores than healthy controls, with symptom severity worsening during active disease and correlating with poorer clinical outcomes. It is also well-documented that many IBD patients experience persistent gastrointestinal symptoms despite the absence of active inflammation, a phenomenon often attributed to overlapping functional gastrointestinal disorders or subclinical disease activity. These symptom-driven states can independently contribute to psychological distress, including anxiety and depression, irrespective of the objective inflammatory burden [5,6]. Therefore, while clinical indices provide a practical method for disease activity classification, they may not fully reflect the complex interplay among inflammation, symptoms, and mental health in IBD patients. Given this bidirectional relationship between disease activity and psychological morbidity, routine screening for mental health disorders is now advocated as part of comprehensive IBD management.

This study aimed to evaluate anxiety and depressive symptom levels in IBD patients, explore associations with clinical and demographic factors, and compare the psychological burden between UC and CD subtypes. Additionally, we benchmarked these findings against a healthy control group to contribute to the evolving literature on IBD–psychiatry comorbidity.

## 2. Materials and Methods

This cross-sectional study included 94 consecutive IBD patients (diagnosed based on standard clinical, endoscopic, and histopathological criteria) under follow-up at the Gastroenterology Department, Trakya University Medical Faculty, Edirne, Turkey and 79 age- and sex-matched healthy controls recruited from routine outpatient clinic visitors.

### 2.1. Participants

- The inclusion criteria for IBD patients were as follows:
  ○ Age ≥ 18 years,
  ○ Confirmed diagnosis of UC or CD.
- The exclusion criteria for IBD patients were as follows:
  ○ Age < 18 years,
  ○ Known psychiatric diagnosis (e.g., major depression, anxiety disorders, bipolar disorder, psychosis)
  ○ Use of psychotropic medication,
  ○ Cognitive or mental impairment precluding comprehension of the questionnaire.

Patients were categorized as newly diagnosed if their diagnosis of ulcerative colitis or Crohn’s disease had been established within the preceding 6 months. Those diagnosed more than 6 months prior to enrollment were considered to have chronic IBD. This classification was used to explore potential psychological differences based on the disease duration.

- The inclusion criteria for controls were as follows:
  ○ Age ≥ 18 years,
  ○ No personal/family history of IBD or chronic illness,
  ○ Absence of cognitive impairment affecting survey comprehension.

Healthy controls were recruited from individuals presenting to the outpatient clinics of Trakya University Medical Faculty for routine health check-ups or non-chronic minor complaints during the study period. A total of 102 eligible individuals were invited to participate, and 79 provided informed consent and completed the study procedures, resulting in an enrollment rate of 77.5%. While the detailed characteristics of non-responders were not systematically recorded, no significant demographic differences were observed based on age or sex at the time of invitation. Controls were matched to the patient group by age and sex to minimize confounding.

### 2.2. Data Collection

Demographic (age, sex, smoking status, medication use, residence) and clinical characteristics (disease subtype [UC/CD], marital status, comorbidities, surgical history, family history of IBD) were recorded. Disease activity was assessed using the following:

- Truelove–Witts index for UC (inactive vs. active [mild/moderate/severe]),
- Crohn’s Disease Activity Index (CDAI) for CD (inactive vs. active [mild/moderate/severe]).

Active disease was also verified using laboratory markers—including hemoglobin, C-reactive protein (CRP), erythrocyte sedimentation rate (ESR), albumin, and white blood cell (WBC) count—as well as endoscopic findings. An endoscopic evaluation was performed for nearly all ulcerative colitis patients and for a limited number of Crohn’s disease patients when clinically indicated, in addition to laboratory and clinical parameters.

### 2.3. Psychological Assessment

Anxiety and depressive symptoms were assessed using the Hospital Anxiety and Depression Scale (HADS), a validated 14-item self-report instrument designed to detect psychological distress in medically ill populations. It consists of two subscales—HADS-A (anxiety symptoms) and HADS-D (depressive symptoms)—each ranging from 0 to 21. While HADS is not intended to establish a formal psychiatric diagnosis, it effectively identifies individuals at risk based on symptom severity. In this study, we applied validated Turkish cutoff values of ≥10 for anxiety symptoms and ≥7 for depressive symptoms [7].

### 2.4. Ethical Considerations

Written and verbal informed consent was obtained. The study adhered to the Declaration of Helsinki and was approved by the Trakya University Ethics Committee (TUTF-BAEK 2016/117), with an approval date of 15 June 2016. Informed consent was obtained from all subjects involved in the study.

### 2.5. Statistical Analysis

Data were analyzed using SPSS 20.0 (IBM Corp.). Continuous variables were expressed as the mean ± SD or median (range), with categorical variables as percentages. Normality was assessed via the Kolmogorov–Smirnov test. Group comparisons used the following:

- Student’s *t*-test (age, normally distributed data),
- Mann–Whitney U test (non-parametric HADS scores),
- Kruskal–Wallis test (HADS scores across UC/CD/controls),
- Chi-square test (categorical variables). A *p*-value < 0.05 was considered statistically significant.
- Given the multiple comparisons starting from Tables 5–10, the false discovery rate was controlled using the Benjamini–Hochberg procedure (q = 0.05). This method was chosen over Bonferroni correction to balance the risk of Type I and Type II errors in this exploratory context. Notably, the associations between active disease and both anxiety and depression scores remained statistically significant following the adjustment, supporting the robustness of our findings.
- Missing data were minimal (<5%) across all key variables. Participants with incomplete HADS responses or missing values for other relevant variables were excluded from the respective analyses using listwise deletion. No imputation methods were applied.

## 3. Results

### 3.1. Participant Characteristics

The study included 94 IBD patients (61 UC [64.9%], 33 CD [35.1%]) and 79 age- and sex-matched controls. Demographic characteristics (age, sex, education, marital status, residence) were comparable between the groups (*p* > 0.05; Table 1). The UC subgroup had a higher proportion of males (62.3% vs. 48.5% in CD), while the control group was balanced (50.6% male) (Table 2). Clinical profiles (symptoms, treatments, comorbidities, surgical history, family IBD history) are detailed in Table 3.

### 3.2. Disease Activity and Psychological Scores

- Disease activity was as follows:
  ○ 45.2% of UC and 48.5% of CD patients were classified as “active” (Table 4).

**Table 4:** Disease activity profiles in UC and CD cohorts.

	Inactive Disease	Active Disease		
		Mild	Moderate	Severe
Ulcerative colitis	41	8	9	3
Crohn’s disease	16	11	6	0

- HADS scores were as follows:
  ○ No significant differences in mean anxiety (HADS-A) or depression (HADS-D) scores were observed among IBD patients (combined), UC/CD subgroups, and controls (*p* > 0.05; Table 5).
  ○ Active IBD patients had significantly higher HADS-A (mean ± SD: 11.2 ± 3.1 vs. 6.8 ± 2.4) and HADS-D scores (8.9 ± 2.7 vs. 4.3 ± 1.9) than inactive patients (*p* < 0.001) and controls (*p* < 0.001).
  ○ Inactive patients showed lower anxiety symptom scores than controls (*p* = 0.005) but comparable depression symptom scores (*p* > 0.05).
  ○ No differences were found between newly diagnosed and chronic IBD patients.

**Table 5:** Hospital Anxiety and Depression Scale (HADS) scores across patient groups and controls.

		Mean	Median	*p*
Controls	HADS-D	5.9367	5.00	0.093
Patients		7.0426	7.00	
Controls	HADS-A	8.2785	8.00	0.360
Patients		8.0957	8.00	
UC	HADS-D	6.8852	7.00	0.204
CD		7.3333	8.00	
UC	HADS-A	7.7705	7.00	0.411
CD		8.6970	8.00	
Active disease	HADS-D	10.0541	10.00	0.000
Inactive disease		5.0870	5.00	
Active disease	HADS-A	10.7027	11.00	0.000
Inactive disease		6.4035	7.00	
Active disease	HADS-D	10.0541	10.00	0.000
Controls		5.9367	5.00	
Inactive disease	HADS-D	5.0870	5.00	1.000
Controls		5.9367	5.00	
Active disease	HADS-A	10.7027	11.00	0.044
Controls		8.2785	8.00	
Inactive disease	HADS-A	6.4035	7.00	0.005
Controls		8.2785	8.00	
New patients	HADS-D	6.25	5.00	>0.05
Old patients		7.0778	7.0	
New patients	HADS-A	6.00	5.50	>0.05
Old patients		8.1889	8.00	

UC: ulcerative colitis; CD: Crohn’s disease; HADS-A: Hospital Anxiety and Depression Scale-Anxiety; HADS-D: Hospital Anxiety and Depression Scale-Depression.

### 3.3. Correlations and Risk Factors

- UC Patients: HADS-A scores correlated with disease activity severity (ρ = 0.42, *p* = 0.003; Table 6).

**Table 6:** Correlation between psychological symptom severity (HADS) and disease activity in IBD patients.

	HADS-D		HADS-A	
	(r)	(*p*)	(r)	(*p*)
Ulcerative colitis	0.212	0.370	0.619	0.004
Crohn’s disease	0.305	0.235	−0.076	0.733

HADS-A: Hospital Anxiety and Depression Scale-Anxiety; HADS-D: Hospital Anxiety and Depression Scale-Depression.

- Education Level: A higher education was associated with lower HADS-D scores (*p* = 0.026).
- Sex Differences: Females had higher HADS-A scores than males (*p* = 0.008; Table 7).

To further evaluate the independent associations with high-risk depression (HADS-D ≥ 7), a multivariate logistic regression analysis was conducted including the disease activity status, sex, and education level. Active disease emerged as an independent predictor (OR: 4.81, 95% CI: 2.01–11.52, *p* < 0.001), as did a lower education level (OR: 1.94, 95% CI: 1.02–3.67, *p* = 0.042). Female sex showed a nonsignificant trend toward an increased risk (OR: 1.47, 95% CI: 0.76–2.84, *p* = 0.250). These findings reinforce the dominant role of disease activity while also highlighting the potential influence of socioeconomic factors on psychological outcomes in IBD.

**Table 7:** Multivariate predictors of anxiety (HADS-A) and depression (HADS-D) scores in IBD patients.

	HADS-D (*p*)	HADS-A (*p*)
Age	0.855	0.937
Gender	0.564	0.008
Education level	0.026	0.718
Marital status	0.967	0.425
Living place	0.102	0.651
Comorbidity presence	0.167	0.144
Disease severity	0.000	0.000

HADS-A: Hospital Anxiety and Depression Scale-Anxiety; HADS-D: Hospital Anxiety and Depression Scale-Depression.

### 3.4. Clinical Risk Stratification Using Validated Cutoffs (HADS-A ≥ 10, HADS-D ≥ 7)

- Depression Risk: This was higher in IBD patients vs. controls (32.1% vs. 17.7%; *p* = 0.028; Table 8).

**Table 8:** Prevalence of high anxiety and depressive symptom burden in IBD patients and healthy controls.

	Control Group (n = 79)	Patient Group (n = 94)	(*p*)
High derpession risk, n (%)	24 (30.4)	44 (46.8)	0.028
High anxiety risk, n (%)	18 (22.8)	25 (26.6)	0.563

- Anxiety/Depression by Subtype: There were no differences among UC, CD, and controls (Table 9).

**Table 9:** Prevalence of high anxiety and depressive symptom burden in ulcerative colitis and Crohn’s disease patients.

	Control Group (n = 79)	Ulcerative Colitis (n = 61)	Crohn’s Disease (n = 33)	(*p*)
High derpession risk, n (%)	24 (30.4)	27 (44.3)	17 (51.5)	0.070
High anxiety risk, n (%)	18 (22.8)	15 (24.6)	10 (30.3)	0.702

- Disease Activity Impact: Active IBD patients had higher risks of anxiety (68.4% vs. 21.1%) and depression (52.6% vs. 12.3%) than inactive patients (*p* < 0.001; Table 10).

**Table 10:** Prevalence of high anxiety and depressive symptom burden by disease activity status in IBD patients.

	Control Group (n = 79)	Inactive Disease (n = 57)	Active Disease (n = 37)	(*p*)
High derpession risk, n (%)	24 (30.4)	16 (28.1)	28 (75.7)	0.000
High anxiety risk, n (%)	18 (22.8)	4 (7)	21 (56.8)	0.000

## 4. Discussion

In our study, we observed that disease activity significantly impacted depression and anxiety scores in IBD patients. Disease activity was a key determinant of both depression and anxiety levels. When evaluating gender differences, while no significant disparity was found for depression, females exhibited higher anxiety scores (*p* < 0.05). Additionally, we identified an inverse correlation between educational attainment and depression scores, suggesting that higher education levels may mitigate depressive symptoms.

IBD typically manifests during a life stage critical for career, family, and social development (ages 15–30), profoundly affecting work, relationships, and emotional well-being [8,9]. The disease’s unpredictable course, debilitating symptoms, frequent hospitalizations, and medication side effects collectively diminish quality of life [10]. Supporting this, a prospective study of 20 IBD patients reported psychological disorders in 60% of patients versus 15% of controls (*p* = 0.009), with anxiety and depression being most prevalent [11]. A meta-analysis of 1,167 pediatric IBD patients further confirmed their elevated depression risk relative to both healthy peers and children with other chronic illnesses [12]. Comparative studies also highlight disparities; the depression prevalence was 29% in Crohn’s disease, 21% in ulcerative colitis, and 11.5% in cystic fibrosis patients (Crohn’s vs. cystic fibrosis: *p* < 0.05) [13].

The chronicity of IBD, coupled with its uncertain progression, surgical risks, and malignancy potential, exacerbates patient anxiety. Anxiety and depression affect 29–35% of patients in remission, escalating to 80% and 60%, respectively, during flares [14]. These findings are supported by large-scale population-based studies such as the Manitoba IBD Cohort Study, in which Walker et al. reported a significantly higher lifetime and 12-month prevalence of anxiety and mood disorders in IBD patients compared to the general population [15]. However, it is important to recognize that psychological distress in IBD is not exclusively driven by measurable inflammation. Previous studies have shown that symptoms such as abdominal pain, diarrhea, and urgency can persist in the absence of active inflammation, leading to significant emotional burdens [5,6]. These findings suggest that functional symptoms, possibly overlapping with irritable bowel syndrome (IBS), may contribute to anxiety and depression independently of inflammatory markers. Since our study relied on clinical indices and did not systematically collect fecal or histological data, it is possible that some patients classified as “inactive” may have had residual low-grade inflammation or functional symptoms that influenced their psychological profile.

Disease severity remains a well-established determinant of psychological morbidity. Brooks et al. identified several risk factors—including disease severity, low socioeconomic status, corticosteroid use, parental stress, and delayed diagnosis—as contributing to mental health vulnerability in IBD patients [16]. Reed-Knight et al. further emphasized the roles of active disease, elevated ESR, and corticosteroid exposure in pediatric Crohn’s-related depression [17], while Szigethy et al. linked active inflammation and steroid use to an increased depression risk [18]. Although our study did not find a significant association between corticosteroid use and psychological scores, disease activity emerged as a strong independent predictor of both anxiety and depressive symptoms (*p* < 0.001), with patients in the active phase demonstrating substantially higher symptom scores compared to those in remission.

Emerging research continues to delineate risk factors for anxiety and depression in IBD. A 1663-patient study associated anxiety with severe flares, treatment nonadherence, disability, unemployment, and socioeconomic deprivation, while depression correlated with age, flares, and similar socioeconomic factors [19]. Gender disparities are also evident; adolescent females with IBD face twice the anxiety risk of males [20], and lower education predicts higher anxiety [21]. Our data align with these trends, showing elevated anxiety in women—likely attributable to societal stressors like caregiving and occupational demands—and reduced depression with higher education, possibly reflecting the benefits of disease literacy and physician counseling. Importantly, a multivariate logistic regression analysis confirmed that disease activity and a lower education level independently predicted high-risk depression, even when adjusted for sex. These findings strengthen the argument that the inflammation-related disease burden and socioeconomic context are key contributors to psychological morbidity in IBD.

The IBD subtype’s role in mental health remains debated. Nordin et al. reported higher anxiety and depression in Crohn’s disease patients (*n* = 492), attributing this to more severe symptoms [22], whereas other studies, like ours, found no subtype-dependent differences [18,23].

## 5. Limitations

This study has several limitations. First, its cross-sectional design precludes causal inference between disease activity and psychological outcomes; while associations are evident, the directionality remains uncertain. Longitudinal studies are needed to confirm whether active inflammation drives psychological morbidity or whether psychological distress contributes to symptom exacerbation. Second, although the total sample size was sufficient to detect major effect sizes—especially between active and inactive disease states—the relatively small Crohn’s disease subgroup may limit the generalizability of subtype-specific findings. Third, although multiple comparisons were made across several hypothesis tests, we applied the Benjamini–Hochberg correction to control the false discovery rate, and key findings remained significant. Finally, the single-center nature of the study may reduce external validity, underscoring the need for multicenter replication.

## 6. Conclusions

The effective management of inflammatory bowel disease (IBD) requires routine assessments and intervention for comorbid psychological conditions. Our findings demonstrate that untreated anxiety and depressive symptoms adversely impact treatment adherence and disease outcomes. Integrating standardized psychiatric evaluations into IBD care through multidisciplinary collaboration is not merely beneficial—it is clinically essential. While our findings underscore the association between clinical disease activity and the severity of anxiety and depressive symptoms, further studies incorporating objective biomarkers and assessments of functional symptoms are needed to clarify the independent contributions of inflammation and symptomatology to mental health outcomes in IBD.

## Figures and Tables

**Table 1:** Demographic and clinical characteristics of inflammatory bowel disease patients and healthy controls.

		Control Group *n* = 79	Patient Group *n* = 94	*p*
Age	Mean ± SD	42 ± 11	41 ± 13	>0.05
Gender, *n* (%)	Male	40 (50.6)	54 (57.4)	
	Female	39 (49.4)	40 (42.6)	
Marital status, *n* (%)	Married	63 (79.7)	70 (74.5)	
	Single	16 (20.3)	24 (25.5)	
Living place, *n* (%)	City	64 (81)	76 (80.9)	
	Rural	15 (19)	18 (19.1)	
Education level, *n* (%)	Primary school	31 (39.2)	42 (44.7)	
	High school and above	48 (60.8)	52 (55.3)	

SD: standard deviation.

**Table 2:** Demographic characteristics by inflammatory bowel disease subtype: ulcerative colitis versus Crohn’s disease.

		Ulcerative Colitis *n* = 61	Crohn ’s Disease *n* = 33
Age	Mean ± SD	43 ± 12	39 ± 15
Gender, *n* (%)	Male	38 (62.3)	16 (48.5)
	Female	23 (37.7)	17 (50.2)
Marital status, *n* (%)	Married	49 (80.3)	21 (63.6)
	Single	12 (19.7)	12 (36.4)
Living place, *n* (%)	City	50 (82)	26 (78.8)
	Rural	11 (18)	7 (21.2)
Education level, *n* (%)	Primary school	27 (44.3)	15 (45.5)
	High school	12 (34.4)	9 (27.3)
	Higher Education School	13 (21.3)	9 (27.3)
Cigarette use, *n* (%)	Yes	13 (21.3)	9 (27.3)
	No	28 (45.9)	14 (42.4)
	Stopped	19 (31.1)	7 (21.2)
	Passive exposure	1 (1.6)	3 (9.1)

SD: standard deviation.

**Table 3:** Baseline clinical characteristics of UC and CD patients.

		Ulcerative Colitis (%)	Crohn’s Disease (%)	*p*
Initial complaint	Abdominal pain	36.1	54.5	0.084
	Diarrhea	52.5	75.8	0.027
	Blood in stool	67.2	18.2	0.000
	Mucous in stool	18.0	12.1	0.455
	Fever	19.7	24.2	0.605
	Fatigue	18.0	27.3	0.296
	Loss of appetite	8.2	24.2	0.031
	Constipation	9.8	3.0	0.230
	Fistula presence	0	27.3	0.000
Medication use	Topical 5-ASA	55.7	0	0.000
	Enteral 5-ASA	73.8	36.4	0.000
	Steroid	9.8	0	0.063
	Azathiopurine	4.9	51.5	0.000
	TNF blocker	8.2	15.2	0.297
	Salazopyrin	3.3	6.1	0.524
	Other	0	3	0.172
Comorbidities	Yes	24.6	18.2	>0.05
	No	75.4	81.8	
Disease-related surgery	Yes	0.00	30.3	<0.05
	No	100	69.7	
Family history of IBD	Yes	14.75	9.09	0.000
	No	85.25	90.91	

5-ASA: 5-aminosalicylic acid; IBD: inflammatory bowel disease; TNF: tumor necrosis factor.

## Data Availability

The original contributions presented in the study are included in the article, further inquiries can be directed to the corresponding authors.
